# Book Reviews

**Published:** 1822-08

**Authors:** 


MONTHLY

### MEDICAL AND SCIENTIFIC BIBLIOGRAPHY.

Vexat censura . . . . . Corvos.

Art. I.—*Observations on Cruritis, or Phlegmasia Dolens.* Communicated in a Letter to John W. Francis, M.D. &c. &c. By David Hosack, M.D.

This letter contains a very short and unsatisfactory account of nine cases of phlegmasia dolens, which have occurred to Dr, Hosack, of New-York, between the year 1791 and the present date. The first which presented itself was treated by cathartics, dilution, and fomentations; but without any regard, we are informed, to the inflammatory character of the complaint. The two next, from viewing the effusion in the limb as bearing an affinity to dropsy, were treated, after active purging, with calomel and squill; one grain of each being administered three times a-day. All these terminated favourably. The fourth case led Dr. Hosack, he says, to a much more enlarged view of the disease. It occurred in a lady who had undergone a severe labour, having borne twins, and suffered much inconvenience from her inordinate size during pregnancy: the lochia had entirely disappeared in ten or twelve days, and the disease came on twenty-three days after delivery. Having been exposed to cold, she was seized with inflammation of the lungs; but, within forty-eight hours, these were entirely relieved by metastasis taking place to the limb. She was bled, purged, took antimonials, and had tepid applications to the part affected; by which means the first stage of the complaint was speedily removed, and the cure afterwards completed by stimulating liniments, friction, and a roller.

The ninth case is the history of a man, who had an affection of the leg and thigh similar to phlegmasia dolens. He was admitted as a fever-patient at the New-York Hospital, in the winter of 1821-22; and in a few days after, he was seized with inflammation in the left leg and thigh, attended with general swelling of the limb, and "exhibiting the remarkable glossy whiteness and œdematous appearance that characterise cruritis in the female, and attended with similar pain and sensibility to the slightest movement." The state of debility in this instance precluded the administration of the usual depleting means, and the disease ended in extensive suppuration, hectic fever, and death.

That phlegmasia dolens is frequently, at its commencement, attended with acute inflammatory symptoms, none who have witnessed the disease will be disposed to deny: nor is it less certain that, after the acute stage has subsided, the lymphatics of the limb show a degree of debility, or more marked disease, which is not met with either in rheumatic or simple inflammatory affections. We were called, last autumn, to a lady, who had been attended, during her accouchement, by our able predecessor. The labour had been natural, and in all respects easy; but, about a fortnight after, she had injudiciously exposed herself to cold by walking in her room without shoes. A few hours after, pains, resembling rheumatism, affected both lower extremities, but soon fixed themselves in the right. Swelling came on, which commenced at the groin, and gradually extended down, occupying principally the inner side of the thigh and leg. Along with these symptoms, there was great constitutional derangement, which required blood-letting and other depleting remedies. By these means the acute symptoms were subdued in a few days; the swelling subsided to a certain extent, and then became stationary; irregular portions of induration marking the course of the lymphatics along the inner side of the limb from the foot to the groin. The application of bandages, friction, and liniments, were had recourse to locally; and, generally, the blue-pill, squills, tonics, and purgatives, were severally tried, under the direction of eminent practitioners, who joined us in consultation; but, to this day, the weakness of the limb, pain, and swelling, remain to a considerable degree. She had been subject to attacks of inflammation of the lungs, and it was the supervention of one of these which rendered free depletion unavoidable. Is it not probable that, but for this circumstance, the sequelæ of the phlegmasia dolens might have been less vexatious?

From his *nine* cases Dr. Hosack is of opinion that the following *twelve* conclusions may be drawn:

"1st. That cruritis is an inflammatory disease, not only affecting the limb, but the whole system.

"2d. That it most usually proceeds from a suppression of the natural excretions, the effect of cold, stimulating drinks, and other means of excitement.

"3d. That it is not necessarily connected with the lochial discharge, as inculcated by Trye, Denman, and indeed by Rodrigus Decastro, of Hamburgh, in 1603, by Wiseman, in 1676, and by Mauriceau, in 1712, who were the authors of this doctrine.

"4th. That the first irritations frequently appear about the calf of the leg, and not in the groins and pelvis, as asserted by Dr. Denman.

"5th. That it follows easy as well as difficult labours, and therefore cannot proceed from the pressure of the child's head upon the edge of the pelvis rupturing the lymphatics, as supposed by Mr. White.

"6th. That it is not a disease confined to the lymphatics, but, as in he cases recorded by Dr. Hull, it appears in every part of the affected limb.

"7th. That it is not confined to females, but, as in the cases recorded by Dr. Hull, Dr. Ferriar, Dr. Thomas, and others, it occasionally appears in males.*

"8th. That, as in gout and rheumatism, when depletion is not actively employed, the inflammation, after appearing in one limb, is in some cases transferred to another.

"9th. That it sometimes appears in both limbs at the same time.

"10th. That the general means of subduing inflammatory action are the most effectual in removing the active stage of this complaint.

"11th. That, in the second stage of cruritis, in addition to the use of general stimuli and tonics, stimulating spirituous liniments, friction, and the roller, are most useful in restoring the circulation, and in exciting the absorbents in the removal of the swelling which remains in the passive stage of this disease.

"12th. That occasionally, as in the cases related by Hull, Denman, and by Zinn, it ends in abscess, and proves fatal, especially where the antiphlogistic treatment has not been vigorously pursued in the first stage of the disease, or when it occurs under great exhaustion and debility of constitution."

---

ART. II.—*Observations on the Use of Emetics in Constipation of the Bowels.* Communicated in a Letter to JOHN B. BECK, M.D. By DAVID HOSACK, M.D.

IN the year 1796, Dr. HOSACK communicated to Dr. PEARSON a remarkable instance of constipation yielding to emetics, after the obstruction had existed for three weeks, which led him to employ the remedy in similar cases. This *ratio medendi* is

* "See *Medical and Chirurgical Journal for* 1817. *Medical and Chirurgical Transactions for* 1819.

founded on the belief that costiveness depends upon an obstructed and torpid state of the liver, which the emetics are intended to remove; "while, by their febrifuge and antispasmodic operation, they are no less useful in removing the fever, the inflammation, and constriction, that constitute some of the most distressing, as well as dangerous, symptoms that attend a constipated state of the belly." Seven cases are related in illustration of this practice, of which the one most in detail, and best suited to our purpose, is that of Miss L——. She has been three times under our author's care for obstinate constipation, being constitutionally inclined to torpor of the bowels. The usual forms of purgatives and purging injections having been administered without effect, an emetic was given, consisting of ipecacuanha and tartarized antimony, by which immediate relief was afforded, although the disease had been present for several days. A second attack was treated in a similar manner, and with similar result. The young lady having suffered a third attack during the illness of Dr. Hosack, she was attended by another physician, who, finding her labour under symptoms of inflammation, with visceral obstruction of seven days' standing, ordered bleeding, with fomentations, glysters, castor-oil, and other laxatives. Finding no relief from these means, the patient, remembering the benefit she had formerly derived from emetics, earnestly solicited a repetition of the same means. This, however, was deemed imprudent by the physician, and recourse was had to calomel and opium, by which salivation was brought on; but the bowels proved obstinate, and refused to move. Fifteen days after the commencement of the obstruction, Dr. Hosack, being sufficiently recovered to visit her, administered an emetic similar to those formerly prescribed, which operated freely, discharging a large quantity of dark-coloured bilious matter by vomiting, and which was followed by similar evacuations by stool. In twenty-four hours, the soreness of the mouth was the only complaint remaining.

From this and similar cases, Dr. Hosack concludes:

"1st. That a constipation of the bowels is usually attended with, and frequently produced by, a torpid state of the liver, and consequent deficiency of the biliary discharge.

"2d. That the pain, spasmodic constriction, and inflammation, attendant upon this disease, are the result either of the mechanical obstruction occasioned by the deficiency of bile, and consequently a retarded peristaltic movement of the intestines, or the effect of a sudden change of perspiration, or of a particular article of diet.

"3d. That, in the commencement of constipation, or in its more advanced stage, when the symptoms of inflammation have been subdued by the lancet, emetics may be very advantageously exhibited, both for the purpose of removing the hepatic obstruction, and of counteracting the spasmodic constriction and pain ordinarily attendant upon this disease.

"4th. That the salutary effects which have been occasionally derived from injections of tobacco-smoke, are attributable to the general relaxation, the nausea, and in some cases the vomiting, which that narcotic produces.

"5th. That the benefits that have in like manner been obtained, in some cases, from the use of tartarized antimony, administered by injection, are to be accounted for by the nausea and vomiting that have been the effects of its operation; but which are to be obtained with more certainty from the same medicine given by the stomach, and to the extent of full vomiting."

It must be quite familiar to every practitioner to see cases of constipation attended with much vomiting: indeed, the irritable state of the stomach is one of those distressing symptoms which we are in the habit of combating by every means in our power. If, therefore, the observations of Dr. Hosack are correct,—we mean, if emetics do really, in a considerable proportion of cases, prove purgative, where other forms of purgatives have failed,—they show that spontaneous vomiting has not the same effect as that artificially produced. In many such examples, every kind of medicine is rejected; and we have known frequent and violent fits of vomiting, for a succession of days, without the slightest tendency to evacuation by the bowels; while they acted as soon as the stomach could be so far assisted as to retain cathartics. Although, therefore, we think the observations of Dr. Hosack of sufficient importance to be kept in mind, yet we cannot but view the plan with some distrust, and would not adopt it without caution.

---

Art. III.—*Observations on Delirium Tremens, or the Delirium of Drunkards; with Cases.* By Dr. Stephen Brown, late resident Surgeon of the New-York Alms-house. (American Medical Recorder, April 1822.)

The author, after enumerating the various names of this malady, as "delirium tremens," "mania a trohi," "febris temulenta," &c. proceeds to tell us at once the object of this essay, —viz. to show the efficacy and safety of opium in its cure, when freely and judiciously administered. The early symptoms are, indigestion, sometimes accompanied with head-ach; trembling of the hands; anxiety of countenance; and delirium, particularly at night. "As the disease progresses," these symptoms, (particularly the delirium,) become more confirmed, and the patients imagine all manner of absurdities: such as, that they hear strange noises, or see rats and mice, or some other creatures, in the room; sometimes they imagine it is the devil, who has come to take them away. During these paroxysms, their appearance and manner are indicative of great terror; and they occasionally console themselves by singing, praying, and repeating passages of scripture.

An idea of the treatment may be gathered from the following sketch of some of the cases. The first related is that of a man forty years of age; he had been in the habit of drinking very freely till within a few days of his attack, when he had omitted all spirituous liquors. In this instance, the patient had been purged with calomel and jalap; he was then bled to the extent of ℥xiv. and had a blister between the shoulders. As the laudanum was not given till after this previous discipline, there is room for scepticism about its effects: he took ʒij. and was much better next day.

Of the second case, we are only told that laudanum was administered "in such doses as to procure sleep," and that the patient did well.

The third case is that of a man, aged forty-eight, who was in the habit of indulging in immoderate potations; and, during one of his drunken fits, had been exposed to intense cold, by which his hands were frost-bitten. As this worthy had been long in the habit of using ardent spirits, according to his own account, to the extent of two or three quarts a-day, it was judged proper to allow him a moderate quantity (we are not told how much,) of his usual stimulus. First night, ʒij. of laudanum: no sleep. Second night, ʒiij.: "in the morning had a little dosing, or imperfect sleep." Third night, ℥ss.: "very little sleep;" spirits discontinued; and, fourth night, ℥ss. of laudanum repeated at bed-time: "next morning, he had not slept." Fifth day, ʒvj. and at the end of an hour ʒij. more, making ℥j. of laudanum within the interval of an hour: slept through that night, most of next day, and following night. After this he was rational, and speedily recovered.

The next case was one of particular urgency, as the patient supposed the devil had come for him; and, accordingly, our author had recourse to doses of opium that might well put his satanic majesty to flight. "I directed ʒj. of laudanum to be given him once an hour until four should be taken, unless sleep should supervene." Sleep did not supervene; and next night the same quantity was repeated; after which, sleep came on, and he became perfectly sane.

The fifth case is the last related by Dr. Brown, and the only additional one of which we mean to take notice: it forms a climax to the whole, and shows to what extent opium may be given, without killing the patient.—M. V. aged forty, a man of robust habit, became affected with delirium tremens. This attack had commenced the day before he was seen by Dr. Brown, who, finding considerable febrile action and a strong pulse, took *twenty ounces of blood from the arm*, applied a blister between the shoulders, and ordered a saline cathartic. Next day, the pulse, &c. was improved, and the Doctor "would have now bled him, but was opposed by the friends;" blisters were applied to the extremities, and nauseating doses of antimony prescribed. Third day, ordered a dose of calomel and jalap; after the operation of which, "a tea-spoonful of laudanum every hour till he should fall asleep." Three drachms were given, when the friends became alarmed, thinking it made him worse, and left it off. From this period little was done till the seventh day, when, the delirium being worse than ever, the following prescription was had recourse to:

℞. Opii, Ipecac. Sodæ sup. carb. ā gr. xxxvj.
Rub intimately, and divide into xij. powders.

One of these powders was ordered every hour till sleep came on. Ten were thus administered, and then the remaining two at once. He vomited a small quantity, fell asleep, and awoke, at the end of twenty-four hours, much better. "He required no more medicine after this, and in a few days went to his daily labour, and has since been as well as formerly."

We have given the practice, and leave our readers to judge for themselves, as we have no room for many comments; but we would just hint that it is not every patient, to whom *thirty-six grains of solid opium* are given in *eleven hours*, who is fortunate enough to vomit a small quantity shortly after. Although it is not mentioned, the probability certainly is, that this "*small* quantity" of ejecta was a *large* quantity of the opium. The practice of administering opium "liberally and judiciously" is not new, although it is new, if not judicious, to give it in such immense doses. Dr. ARMSTRONG recommends about forty or fifty drops of laudanum to be given in warm wine, and repeated at the interval of two or three hours, if sleep has not rendered it unnecessary; but he cautions us, "that it is very perilous practice to administer it in too large and repeated doses; since apoplexy, coma, or convulsions, may be thereby produced."

---

**ART. IV.—*Pratique d'Accouchemens; ou Mémoires et Observations sur les Points les plus importans de l'Art.* Par Mad. LACHAPELLE, Sage-femme en chef de la Maison d'Accouchemens de Paris. Publiée par M. ANT. DUGÈS, son Neveu, Docteur en Medecine. 1 vol. 8vo. 1821.**

To recommend this work to attention, it is enough to mention that the author, who was regarded by the faculty in Paris as a very intelligent person, practised her profession for thirty years, and was present at more than 40,000 labours.

Madame LACHAPELLE's work consists of an introduction, containing an account of the Maternité, and three memoirs. In the first of these, she treats of the positions of the fœtus generally, considering them with regard to number, frequency, diagnosis, prognosis, cause, indication, and the means to be employed. Into these it is impossible for us to follow her. She thinks BAUDELOCQUE has enumerated a greater variety of positions than really occur,—at least, than she has ever met with during her very extensive observations. Some remarks are occasionally made, the accuracy of which we are much inclined to doubt; such as, that fracture of the cranium is no very are occurrence in spontaneous and simple labours.

In the second memoir, she treats of the position of the vertex; under which name she includes the entire occiput and sides of the head. She admits six kinds of position, four oblique and two transverse; from these originate many varieties, which render the new nomenclature complicated. This memoir is followed by no fewer than eighty-six observations.

The third relates to the positions of the face; under which title are comprehended all the parts between the ears, the fontanelle, and the larynx. To this are attached thirty-seven observations; but our limits do not permit us to enter into the subject more minutely, and we must refer those interested in it to the book itself.

---

ART. V.—*Anatomie de l'Homme, &c.* Par JULES CLOQUET.

THIS work has already been alluded to in our Journal; but the arrival of some additional Numbers induces us to call the attention of our brethren more particularly to it.

Livraison I. contains general considerations on the Structure of the Human Body; and then proceeds to the Bones in particular, treating of the skeleton, the joints, the trunk, the articulations of the vertebral column, and the vertebral column in general. The 1st Plate contains various figures illustrative of these subjects; consisting of portions of bone, muscle, nerve, blood-vessel, &c. &c. &c. The 2d, 3d, 4th, and 5th, different views of the spinal column. The 6th is a view of the ribs and sternum.

Livraison II.—Plate 7th, back view of the chest; 8th, the sternum and ribs separately; 9th and 10th, the ribs in their natural situation, externally; ditto, internally; 11th, a variety of figures, showing the ligaments about the sternum, and between the vertebræ and ribs; 12th, the frontal bones of various ages, and exhibited in different positions.

Livraison III.—Plate 13th, the parietal and occipital bones in different positions; 14th, the temporal and ethnoid bones in a great variety of views; 15th, sphenoid of an adult, natural size, in different views; ditto of fœtus at the full period; 16th, the occipital bone of a fœtus at the full period; the ethnoid ditto; the parietal ditto; ossa wormiana, and cranium of a woman thirty-six years of age, seen from above, natural size; 17th, profile of the cranium without the bones of the face; view of the base of the skull, natural size; 18th, vault of the cranium, and inside view of the base of ditto.

Livraison IV.—Plate 19th, various views of the upper jaw-bone, os molæ, and os unguis; 20th, the palate bone, vomer and ossa turbinata; 21st, various views of the lower jaw-bone; 22d, both jaws, with the teeth *in situ* and separately; 23d, shows the formation of the teeth in the jaw; 24th, articulation of the jaw, entrance of the submaxillary nerve, &c.

Livraison V.—Plate 25th, a front and back view of the skull and lower jaw, of natural size; 26th, view of different sections of the skull in profile; 27th, ditto of the upper jaw, showing the antrum, &c. crania of fœtuses; 28th, the cranium of a woman aged ninety years, illustrating the changes produced by time; various illustrations of the facial angle, taken from the heads of Bichat, the Hottentot Venus, an orang-outang, and a wolf; 29th, heads illustrating the peculiarities of different races; 30th, ditto, ditto.

Livraison VI.—Plate 31st, various articulations of the vertebræ of the neck; 32d, os sacrum, os cocygis, and ossa innominata; 33d, back and front view of the pelvis; 34th, various views of ditto; 35th, ditto with ligaments; 36th, pelvis of fœtus, adult scapulæ, clavicles, and patella.

Livraison VII.—Plate 37th, views of the os humeri and radius of adults, ditto of fœtus, ditto of articulating extremities; 38th, the ulna and bones of the carpus; 39th, the bones of the metacarpus and fingers; 40th, articulations of the clavicles and first ribs with the sternum, of the clavicle with the scapula, of the humerus with ditto, showing the connecting ligaments; the glenoid cavity and ligaments; 41st, the anatomy of the elbow joint, the inter-osseous membrane, the joint of the wrist; 42d, ligaments connected with the wrist, carpus, and fingers; section of the bones of the finger; the articulation of the radius, with the upper extremity of the ulna.

Livraison VIII.—Plate 43d, thigh bone of adult and fœtus, articulating extremities of ditto, patella; 44th, tibia and fibula of ditto, with ditto; 45th, bones of the tarsus separately; 46th, ditto of metatarsus and toes separately; 47th, various views of the foot; 48th, ligaments, hip and knee joints.

We have been thus particular in our enumeration of the contents of the different parts of M. Cloquet's work, because, each livraison being sold separately, our readers will thus be enabled to procure any plates they may want. The manner in which these are executed, and the copious text by which they are accompanied, render the work an important addition to our anatomical books; while the price is comparatively so moderate as to put it in the power of many to procure them, who would not think of purchasing plates of the same kind executed in this country.

The utility of this work is greatly enhanced by the difficulties attending a course of dissections any where except in great towns; and, although we should be the last to recommend that the study of plates should be substituted for that minute and practical acquaintance with structure and the relative situation of parts which can only be acquired in the dissecting room, yet, on the other hand, we know that many practitioners in the country, who have acquired their knowledge in this legitimate way, would find great satisfaction in being able to recal those impressions which, unavoidably, become less vivid and distinct when they have been long removed from the practical means of refreshing them. Nay, even in London, we are convinced that many surgeons, in the prospect of performing an operation for strangulated hernia, or tying a great artery, would find it no way unpleasant to have looked over the delineations of the parts in such a work as Cloquet's. A great drawback to such plates in general (besides their expense,) is, that they are executed either by anatomists who are not draughtsmen, or by draughtsmen who are not anatomists. Many artists, it is true, talk of having studied anatomy; but then it is only what may justly be called superficial, being literally confined to the surface, the angles of bones, and prominence of muscles, by which the contour of the figure, or expression of the countenance, may be accurately delineated. In making these remarks, it is but justice to Mr. CHARLES BELL to point him out as an exception; but, on the other hand, he is a solitary instance, in this country, of an able draughtsman and practical anatomist united in one person. CLOQUET, we are assured, is himself expert at designing, and M. HAINCELIN and M. FEILLETTE, his assistants, have bestowed particular attention to anatomy; circumstances which account for the neatness, precision, and accuracy, which distinguish these plates. We shall continue our *Index* from time to time, for the reasons above mentioned.

---

ART. VI.—*The Naturalist's Repository, or Monthly Miscellany of Exotic Natural History; consisting of elegantly coloured Plates, with appropriate, scientific, and general Descriptions of the most curious, scarce, and beautiful Productions of Nature, &c. &c. (To be continued Monthly.)* Simpkin and Marshall, London.

WE have before us the two first Numbers of this work, each of which contains three well-finished and coloured engravings, illustrating subjects of conchology, entomology, and ornithology, with suitable scientific descriptions.

Natural history is an important branch of medical science; but the constitution of our schools is so unfavourable to its cultivation, that its study is generally neglected. It is much to be regretted that, while our great continental rival abounds with myriads of highly-preserved specimens of natural science, accessible to every student, we are left, in this country, with little resource but such as we can derive from publications like that of

Mr. DONOVAN'S. The author promises the completion of the work in sixty Numbers; and, judging from the commencement of his undertaking, we anticipate an interesting compendium of all that is valuable on the subject which it professes to treat.

---

ART. VII.—*Appendix to the General System of Toxicology, or a Treatise on Mineral, Vegetable, and Animal Poisons.* By M. ORFILA, Professor of Medical Jurisprudence in the University of Paris, &c. &c. &c. *Containing all the additional Matter relating to that Science published by the Author in his late Work, entitled, Lectures on Medical Jurisprudence, and thus rendering complete the former Treatise on Poisons. To which are added, Twenty-two coloured Engravings of Poisonous Plants, Fungi, Insects, &c.* Translated from the French, by JOHN AUGUSTINE WALTER.

THE original "Leçons de Medecine Legale," of Professor ORFILA, have been so fully reviewed in three succeeding Numbers of our Journal, for July, August, and September, 1821, that any detailed account of the present translation of a part of them would be superfluous.

Although toxicology necessarily forms a part of a system of medical jurisprudence, it is also a distinct branch of science, and, as such, has been separately treated by the author. Subsequently to the publication of the work on Poisons, the author, availing himself of recent discoveries in the analysis of vegetable substances, has pursued his investigations, and has incorporated their results in the above work. Mr. WALTER has made a useful selection of so much as is necessary to complete the former, which he gives under the title of an Appendix. This little work contains, in a narrow compass, much useful information; and its value is enhanced by the addition of numerous highly-finished engravings of poisonous plants and insects.

---

ART. VIII.—*De la Medecine Operatoire;* par R. B. SABATIER, Chirurgien en chef de l'Hotel des Invalids, Membre de la Legion d'Honneur, de l'Institut de France, et de plusieurs Academies, &c. Nouvelle Edition, faites sous les yeux de M. le Baron DUPUYTREN, Membre de la Legion d'Honneur, de l'Ordre de Saint Michel, Chirurgien en chef de l'Hotel Dieu, &c. &c. Par L. J. SANSON, Docteur en Chirurgie, &c. et L. I. BEGIN, Chirurgien Aide-Major à l'Hôpital Militaire, &c.—Imported by Burgess and Hill.

A WORK from the pen of Professor SABATIER is above all praise,—least of all does his Operative Surgery require any eulogy from us: a period of twenty-six years and upwards, since its first publication, have enabled the profession to appreciate the merits of this classical production, which, notwithstanding some defect in the arrangement of the first edition, was, and still is, the most complete, the most luminous, and most valuable epitome of modern surgery. This branch of medical science has made incontestible progress since the last edition; a new one, therefore, containing the recent improvements, was a desideratum; and we cannot have a greater earnest of the advantageous manner in which the present has been prepared, than the respectable names of the two editors, who have executed their task under the eyes of one of the most distinguished professors of this or any age.

A work of this kind obviously defies all attempt at useful analysis, without the quotation of the whole. It is, in itself, an analysis of all that is valuable relative to the various operations in surgery.

The text of the last edition has been studiously preserved: where the editors have had occasion to differ from the author, they have availed themselves of notes for the expression of their sentiments. Those additions which have been rendered necessary in consequence of the progressive improvements in the science, have been incorporated with the text; but, that the reader may be able to distinguish between the original and superadded matter, the latter is included between brackets. Among the additions we find, partial amputations of the bones of the foot; operation for cataract, by Keratonyxis; aerian fistulæ; hydropericardium; panaris; carcinoma; prolapsus of the rectum; internal strangulation; artificial anus; ligature of the axillary, subclavian, carotid, and external iliac arteries; and of the trunk itself of the abdominal aorta.

The most considerable addition occupies nearly 400 pages of the first volume, under the general head of "Prolegomenes," in which the minor operations of surgery are given, such as bleeding, arteriotomy, scarification, acupuncture, dry and sanguineous cupping, the application of seton, caustics, leeches, and moxa, or other substances in combustion, as phosphorus, gunpowder, &c. cauterization by the rays of the sun or of fire, &c. In an article of some length, the subject of burns is admirably treated. All these were omitted by Sabatier, although it must be confessed that they are no less deserving of notice than the capital operations. The practical surgeon, who has already acquired this information from actual observation, does not stand in need of books of reference; but the student will find his path considerably smoothed by the perusal of this part of the work.

The anatomical descriptions of the parts subjected to operation,—the time best suited to its performance,—the local and constitutional treatment afterwards,—the preparation of the patient,—the suppression of hemorrhage,—and a variety of similar considerations, occupy a portion of the author's attention.

